# Commentary: Oscillatory Neuronal Activity Reflects Lexical-Semantic Feature Integration within and across Sensory Modalities in Distributed Cortical Networks

**DOI:** 10.3389/fpsyg.2015.02005

**Published:** 2016-01-06

**Authors:** Svetlana Pinet, Raphaël Fargier

**Affiliations:** ^1^Laboratoire de Psychologie Cognitive, Aix-Marseille UniversitéMarseille, France; ^2^Faculté de Psychologie et des Sciences de l Éducation, University of GenevaGeneva, Switzerland

**Keywords:** MEG, cortical oscillations, semantic integration, features verification, semantic hub, sensory modalities

## Introduction

Semantic knowledge relies on widely distributed brain networks. According to embodied semantics, retrieving features associated to words reactivate the sensory systems used during the encoding of the objects or entities they refer to Barsalou (2008). This does not preclude that semantic knowledge is organized hierarchically from modality-specific brain regions to higher-level, “convergence zones” or “hubs” (Meyer and Damasio, 2009). This organization could provide the necessary dynamic neural network for flexible lexical-semantic representations (Willems and Casasanto, 2011), although it could alternatively be interpreted in favor of amodal abstract representations (Mahon, 2015). Nevertheless, to what extent are these regions differentially activated and functionally participate to context-directed semantic integration? Eventually, which parts of this network are fundamentally modal, cross-modal, or amodal, and what is the specific role of the anterior temporal lobe (ATL), remains an area of debate.

## Paradigm and main results

In a recent study published in the Journal of Neuroscience, van Ackeren et al. (2014) attempted to determine whether words are processed differently when read in the context of words strongly associated with particular sensory modalities. Authors used magnetoencephalographic (MEG) recordings and a dual property verification task, in which two feature words were followed by a target word. Feature words either referred to a single modality (modality-specific or MS, e.g., “red,” “big”; i.e., vision), or different modalities (cross-modal or CM, e.g., “red,” “loud”; i.e., vision/audition). The subject's task was to evaluate if feature words were appropriate descriptors of the target.

Van Ackeren et al. (2014) focused on oscillatory activity across a wide range of frequencies (from 2 to 150 Hz) during the processing of the target word. In the high frequencies (gamma range: 80–120 Hz), early enhanced power (150–350 ms) was observed for the MS>CM contrast in a cluster of occipito-temporal sensors. In contrast, in the low frequencies (theta range: 2–8 Hz), late effects (580–1000 ms) were found with enhanced power for CM>MS primes in a cluster of left-lateralized sensors.

## Discussion

First of all, the authors' underlying hypothesis is that if a target word automatically activates all of its associated semantic features, the prior integration of feature words, whether MS or CM, should not influence the processing of the target word. However, the theoretical assumption associated with this paradigm—that target word processing reflects time-locked integration of feature words—needs to be carefully handled. In such a paradigm, feature words are most probably being processed during their presentation. This supposedly activates different networks, in turn influencing the processing of a subsequent word. Although enhanced theta oscillations in CM condition might reflect residual far-reaching connections between areas (van Ackeren and Rueschemeyer, 2014), what is going on within these distributed networks as feature words are being integrated with each other is kept aside. One could already expect differences, whether evoked or induced, related to the second feature word presentation and whether both words refer to the same or different modalities (e.g., N400, Kutas and Federmeier, 2011).

The main result discussed in van Ackeren et al. (2014) is that opposite gamma and theta effects both originate in left ATL. It suggests ATL sensitivity to cross-modality, which is consistent with its anatomical connections to sensory areas. However, ATL present fine-grained subdivisions, whose function might differ (Bonner and Price, 2013). Which part of the ATL is functionally involved in combining specifically auditory-visual information relative to general multimodal semantic information thus remains to be clarified. Accordingly, how do these results relate to the “amodality” of the ATL? An amodal hub should be insensitive to any modality of a word's features (haptic, olfactory, etc.) and to stimulus input modality (pictures, sounds, etc.), which was not tested here. In this regard, ATL but also the precuneus (Fairhall and Caramazza, 2013), the posterior part of MTG, the angular gyrus (Binder et al., 2009), or the right ATL (Jefferies, 2013) are all valid candidates. Moreover, an amodal hub should present the ability to generalize across several instantiations of the same concept. This implies connecting with other brain areas to form a dynamic network within which relevant core features will be extracted. This “neuronal assembly” view for memory traces clearly contrasts with the task at hand, dealing with on the spot features “integration”, i.e., with integrating features that *can be* related to the target concept but are probably not its *core* features. Still, van Ackeren et al. propose two distinct mechanisms for combining information, according to the modality(ies) they relate to. This promising mechanistic framework could well underlie new features learning.

However, any hypothesis about network dynamics falls short, as authors did not explicitly study interactions between the involved brain regions. Effective connectivity analyses could shed light on the organization of the semantic network, from sensory systems toward potential hubs. If the ATL is indeed a “convergence zone,” then its activity should be highly related to “features zones” (Patterson et al., 2007; Coutanche and Thompson-Schill, 2014). In particular, authors report similar gamma activity between sensory areas and ATL. As information is progressively being processed and integrated, activities should become coherent, which could be assessed via Phase-Locking Value or any other coherence measure. Such analyses could also help determining the direction of the late theta effect: integration from sensory systems—retrieving the concept—or reaching out to other brain networks—activating other features (McNorgan et al., 2011). Moreover, measures of cross-frequency phase-amplitude coupling in the ATL could add valuable insight on the relationship between the effects reported in gamma and theta frequency bands (see Canolty et al., 2006).

Van Ackeren et al.'s study of oscillatory patterns within and across neural networks provides precious evidence on the functional dynamics of semantic integration. In particular, it proposes an interesting mechanistic framework for semantic integration through oscillatory modulations. Studying synchronization between putative hubs and features zones could potentially enrich the embodiment debate. Then, to what extent the mechanistic differences observed can be reconciled with amodal properties of ATL still needs further investigation. The similar integration of feature names and features *per se* should also be examined to disentangle lexical-semantic from multi-sensory processing and shed light on representations content.

## Funding

This work was supported by a doctoral MNRT grant from the Ministère de l'Enseignement et de la Recherche (France) and by the Swiss National Science Foundation (grant number 105319_146113).

### Conflict of interest statement

The authors declare that the research was conducted in the absence of any commercial or financial relationships that could be construed as a potential conflict of interest. The reviewer Kristof Strijkers declared a shared affiliation, though no other collaboration, with one of the authors Svetlana Pinet to the handling Editor, who ensured that the process nevertheless met the standards of a fair and objective review.
